# Supplementary material for: SCL14 Inhibits the Functions of the NAC043–MYB61 Signaling Cascade to Reduce the Lignin Content in Autotetraploid Populus hopeiensis
Source: Int J Mol Sci. 2023 Mar 18;24(6):5809. doi: 10.3390/ijms24065809 (PMC10051758; doi:10.3390/ijms24065809)
Supplement: Supplementary file 1 [file ijms-24-05809-s001.zip › Supplementary Figures.pdf]

# Supplementary Figures:

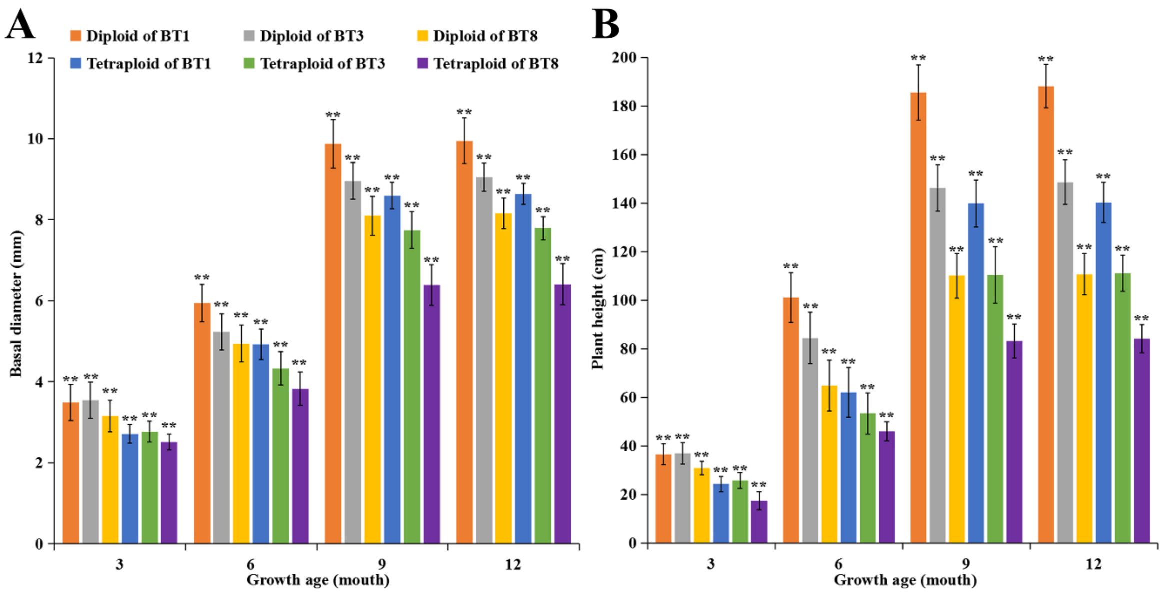


**Figure S1.** Basal diameter (**A**) and tree height (**B**) of 3-, 6-, 9-, and 12-month-old diploid and tetraploid plants of *P. hopeiensis*. The vertical bars show the standard error; the asterisk indicates significant differences between diploid and tetraploid plants (* $p < 0.05$, ** $p < 0.01$).

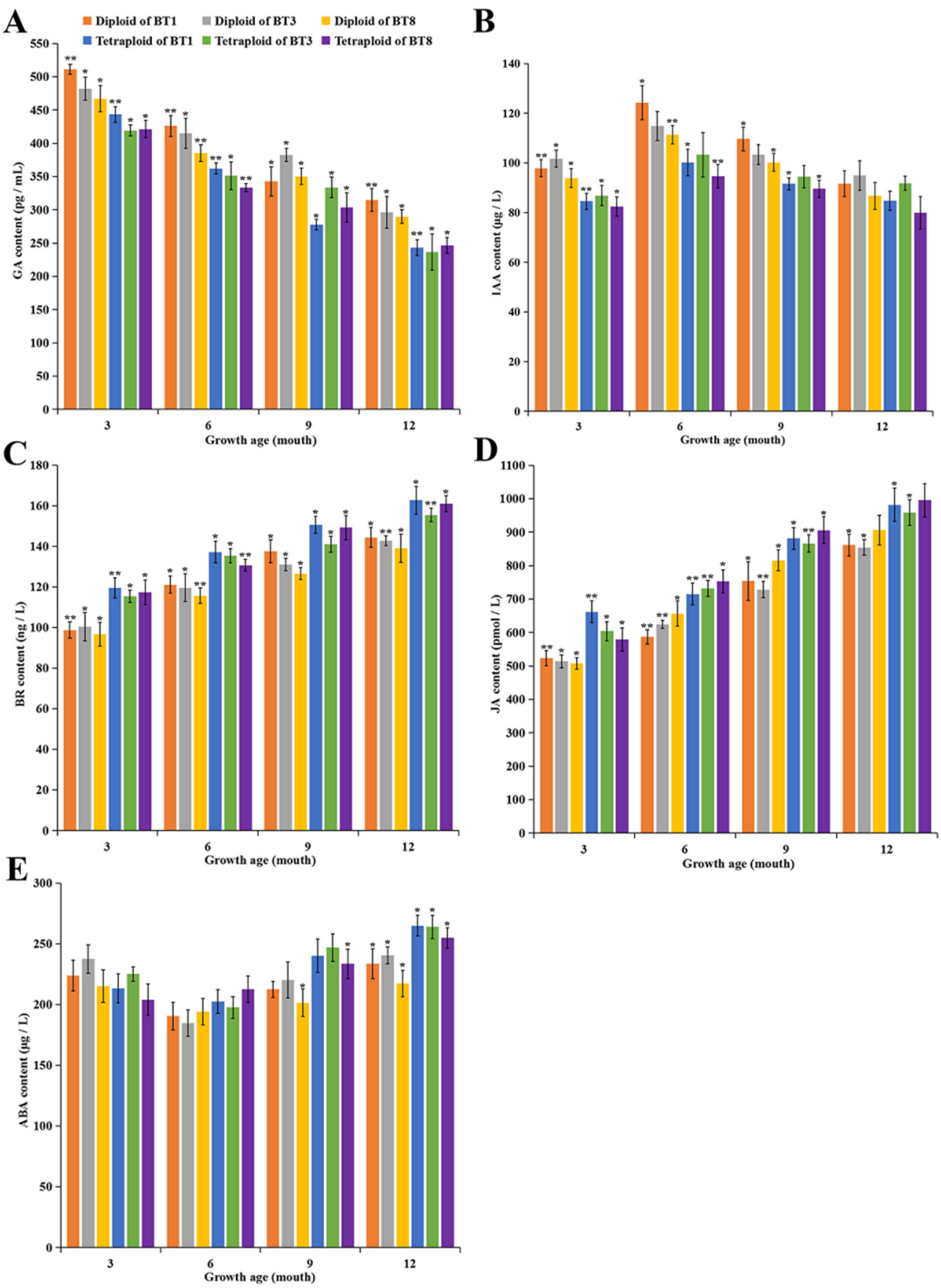


**Figure S2.** The content of GA (**A**), IAA (**B**), BR (**C**), JA (**D**), and ABA (**E**) of 3-, 6-, 9-, and 12-month-old diploid and tetraploid plants of *P. hopeiensis*. The vertical bars show the standard error; the asterisk indicates significant differences between diploid and tetraploid plants (* $p < 0.05$, ** $p < 0.01$).

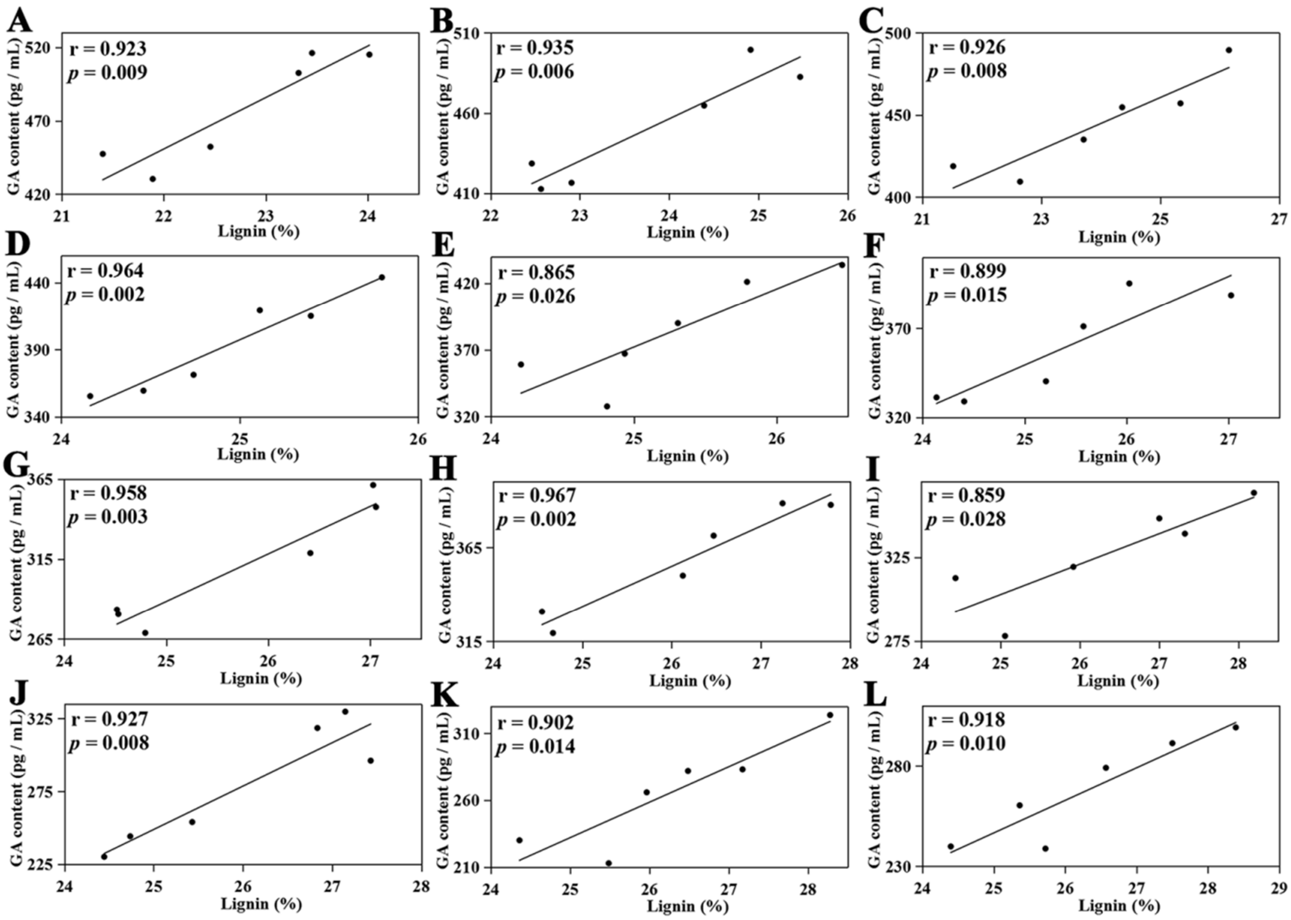


**Figure S3.** Phenotypic correlations between the GA content and lignin content of each clone at all developmental stages. (**A**, **B**, and **C**) Phenotypic correlations between the GA content and lignin content of 3-month-old diploid and tetraploid plants of clones BT1, BT3, and BT8, respectively. (**D**, **E**, and **F**) Phenotypic correlations between the GA content and lignin content of 6-month-old diploid and tetraploid plants of clones BT1, BT3, and BT8, respectively. (**G**, **H**, and **I**) Phenotypic correlations between the GA content and lignin content of 9-month-old diploid and tetraploid plants of clones BT1, BT3, and BT8, respectively. (**J**, **K**, and **L**) Phenotypic correlations between the GA content and lignin content of 12-month-old diploid and tetraploid plants of clones BT1, BT3, and BT8, respectively.

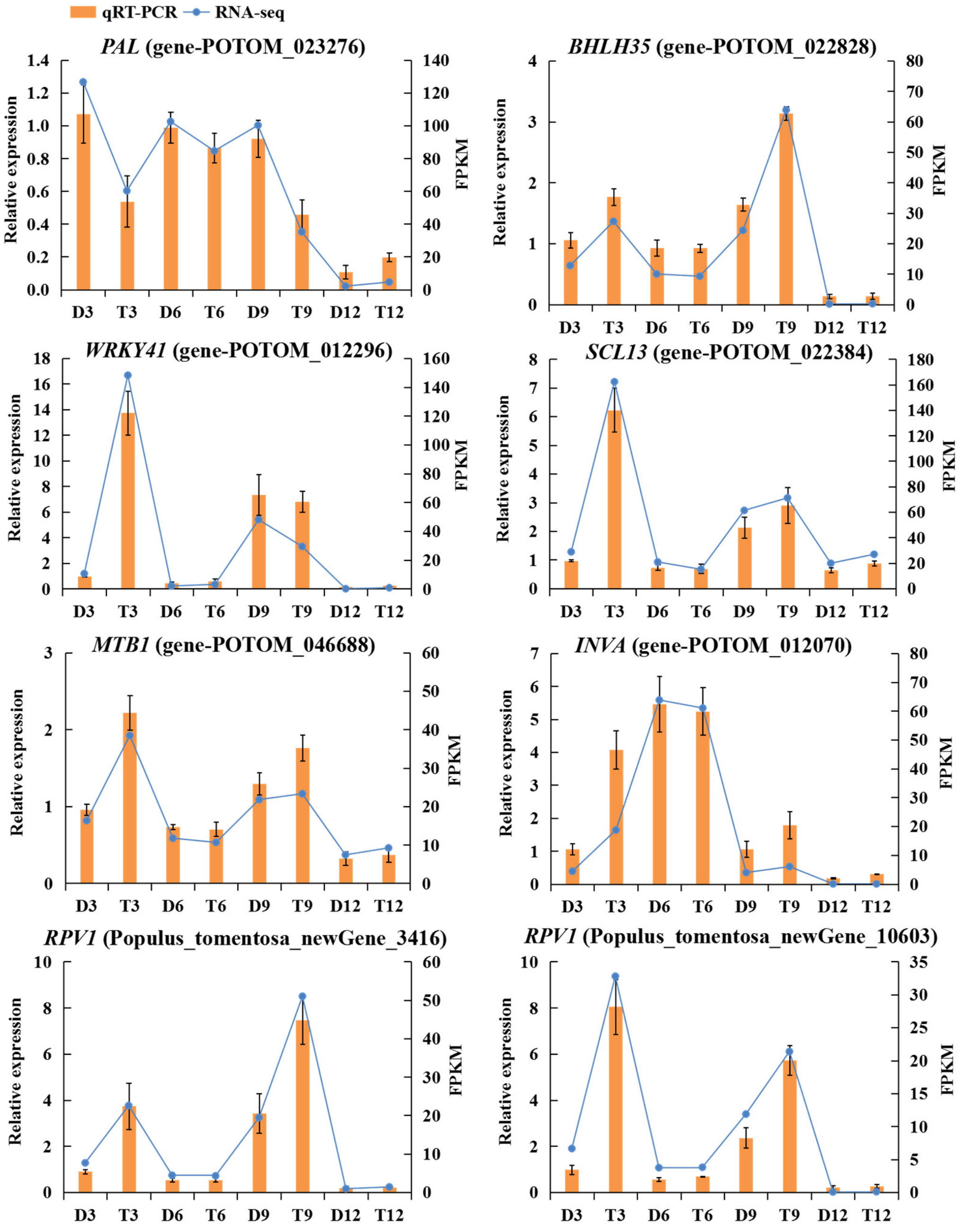


**Figure S4.** qRT - PCR verification of expression level of eight DEGs identified by RNA sequencing. The *y*-axis on the left indicates the relative gene expression levels ($2^{-\Delta\Delta CT}$) analyzed by qRT-PCR, while the *y*-axis on the right represents the FPKM value obtained by RNA-seq. The *x*-axis represents the diploid and tetraploid samples at different developmental stages.

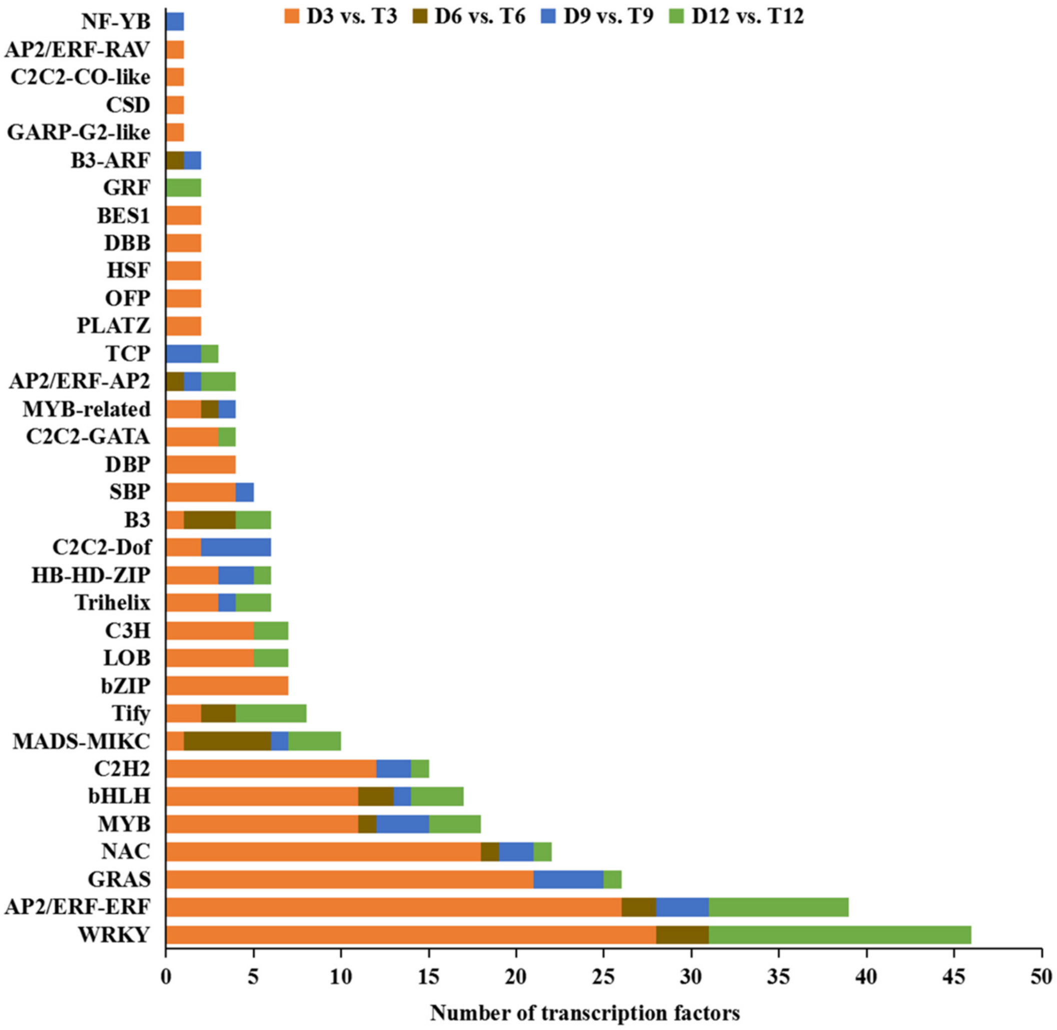


**Figure S5.** The distribution of differentially expressed TFs in diploids and tetraploids at different developmental stages.

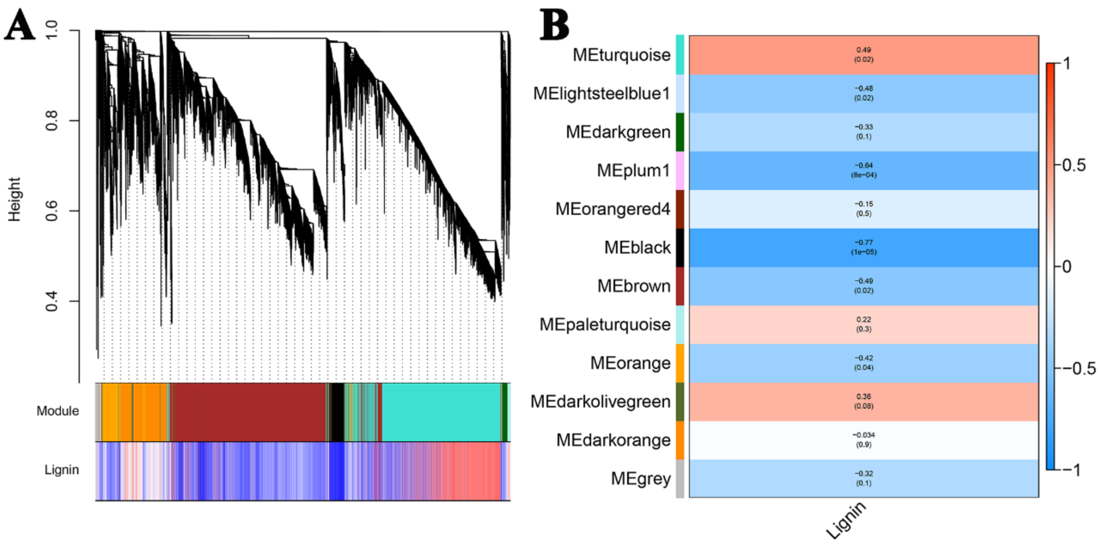


**Figure S6.** Co-expression analysis of all expression genes and lignin contents in *P. hopeiensis*. (**A**) Clustering dendrogram of all expression genes. (**B**) Correlations of module and lignin contents with corresponding *p*-values.

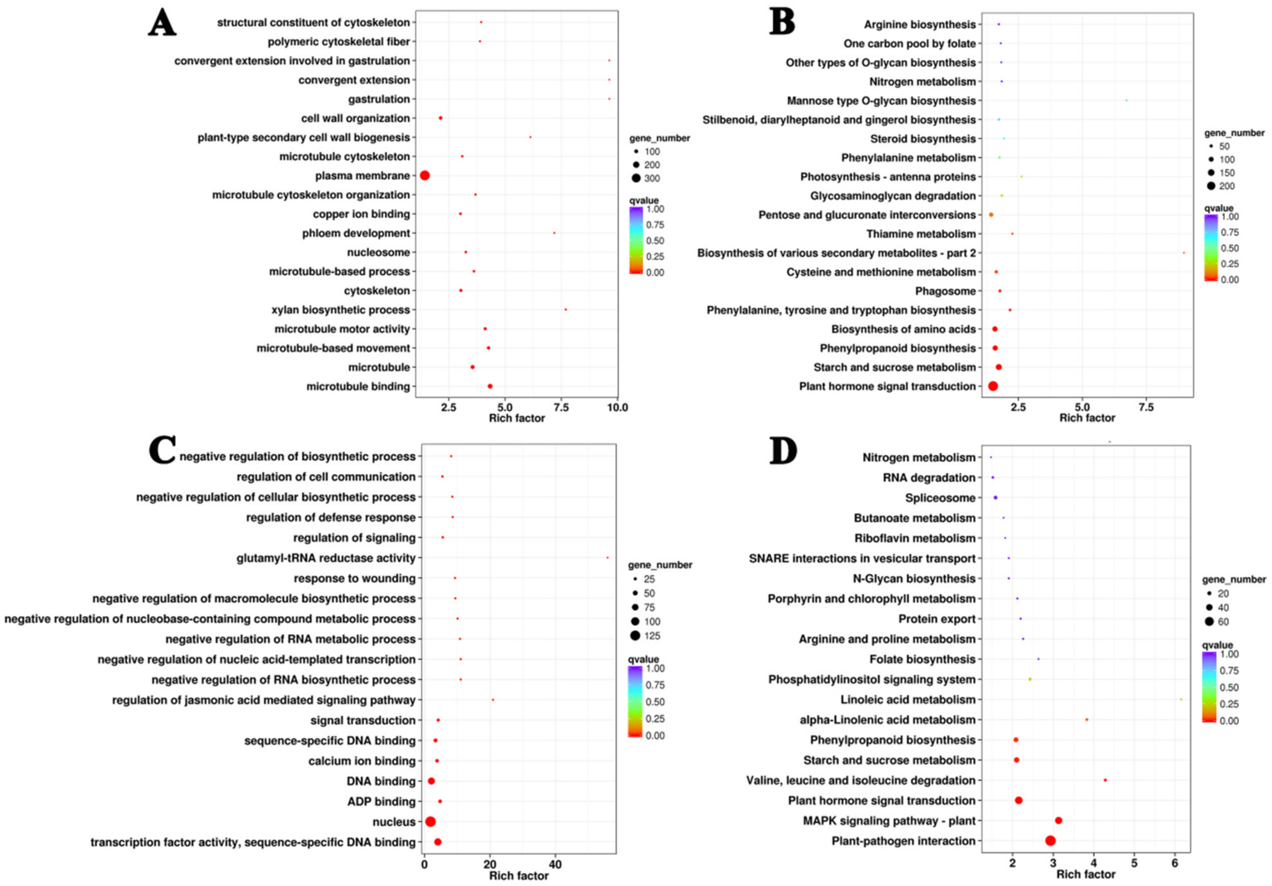


**Figure S7.** GO enrichment (**A**) and KEGG enrichment (**B**) analysis of all genes in brown module; GO enrichment (**C**) and KEGG enrichment (**D**) analysis of all genes in orange module.
